# Observations on Diseases in the Bristol Dispensary

**Published:** 1801-09

**Authors:** W. D. Rolfe

**Affiliations:** Dispensary House, North Street


### *To the Editors of the Medical and Phyfical Journal.*

Gentlemen,

In the catalogue of difeafes which generally prevail and afflict the moft indigent clafs of this city are thofe of the infectious kind, particularly Putrid and Typhus Fevers. The extreme diftrefs under which the poor have lately laboured, has not only been conducive to the propagation of this difeafe, but alfo to its malignity; and at the fame time it has operated as a great obftacle to the cure. However, I flatter myfelf that I have been as fortunate in the treatment of them as I could expect; and a great number having been placed under my care, I have had a fufficient opportunity of confirming the general efficacy of the medicine I have employed.

The ufe of yeaft, whenever I had a fair opportunity of trying it, proved very fuccefsful; but I have experienced many difappointments in general with it, owing to the obftinate prejudice of the indigent againft any thing they fuppofe themfelves familiar with, and capable of judging about. If the patients are willing to take it, the vifitors, friends, and neighbours will foon, without farther inveftigation, prejudice them againft it.

In confequence of thefe accidental inconveniencies, I have endeavoured to adopt a treatment as cheap as eligible, and as agreeable to my fituation; after five months trial, I found its effects to anfwer my moft fanguine expectations. The refult of of it was, in that time I admitted 211 Typhus caſes, out of which 11 died.

My treatment was as follows: In the firſt ſtate of the fever I univerſally gave an emetic; then the following mixture, ℞. Acid. muriat. ʒj. Syr. ſimp. ℥ſs. Decoct. cinchonæ flavæ ℥ij. Aq. puræ q. s. ft. miſt. capt. coch. iij. amp. 4tis horis; and in the more advanced ſtate I gave the following, ℞. Acid. muriat. ʒj. Decoct. cinchonæ flavæ ℥viij. M. capt. coch. iij. amp. 4tis. horis.

*Diſpenſary Houſe, North Street,*

*Auguſt* 10, 1801.

I am, &c.
W. D. ROLFE.

