# Errata

**Published:** 1801-10

**Authors:** 


### ERRATA.

Vol. VI. p. 197, l. 5, *for* ℥j. muriatic acid, *read* ʒj.

